# High resolution physically based modelling reveals malaria incidence reduction by vector control measures

**DOI:** 10.1038/s41598-025-33539-w

**Published:** 2026-01-08

**Authors:** Mame Diarra Bousso Dieng, Stephan Munga, Adrian M. Tompkins, Miguel Garrido Zornoza, Cyril Caminade, Benjamin Fersch, Joël Arnault, Sammy Khagayi, Maximilian Schwarz, Simon Kariuki, Godfrey Bigogo, Harald Kunstmann

**Affiliations:** 1 Karlsruhe Institute of Meteorology, Campus Alpine, Kreuzeckbahnstrasse 19, 82467 Garmisch-Partenkirchen, Germany; 2 Kenya Medical Research Institute (KEMRI), Kisumu, Kenya; 3 International Centre for Theoretical Physics (ICTP), Trieste, Italy; 4 Institute of Geography, University of Augsburg, Augsburg, Germany; 5 Remote Sensing Solutions GmbH (RSS), Munich, Germany; 6 Center for Climate Resilience, University of Augsburg, Augsburg, Germany

**Keywords:** Malaria transmission dynamics, Model coupling and optimization, Health and demographic surveillance systems, Bet net use, Climate sciences, Diseases, Ecology, Ecology, Environmental sciences

## Abstract

Malaria continues to cause over 600,000 deaths annually in sub-Saharan Africa, disproportionately affecting children under five. Despite sustained control efforts, transmission remains highly sensitive to local environmental and climatic variability, underscoring the need for physically grounded models capable of capturing these dynamics. To address this challenge, we developed a high-resolution hybrid modeling framework linking WRF/WRF-Hydro and VECTRI. The framework integrates atmospheric, hydrological, ecological, and intervention processes at 1 km and 50 m resolutions and includes a new compartment for insecticide-treated net (ITN) coverage. Using data from 2007–2022 in western Kenya, a period of large-scale ITN deployment, the model reproduced observed malaria trends with a mean monthly deviation of ±100–150 cases. Simulations showed that ITN coverage reduced the entomological inoculation rate and malaria incidence by 58% and 41%, respectively, with the highest efficacy under warm ($\approx 29^\circ$C) and moderately wet (150–250 mm) conditions. The findings suggest that integrating environmental process modeling with optimized, targeted control strategies provides a cost-effective and operationally relevant framework for sustainable malaria management under changing climatic conditions.

## Introduction

Malaria remains a pervasive and devastating public health challenge, particularly in endemic rural areas of Sub-Saharan Africa (SSA), where its socioeconomic burden is disproportionately high. Between 2000 and 2024, malaria represented approximately 21% of outpatient consultations and 20% of inpatient admissions, with some countries reporting figures as high as 70%^1^.

In Kenya, malaria alone represented approximately 15% of outpatient consultations in 2022^2^ with an estimated 3.3 million malaria cases in 2023^1,3^. However, counties with high transmission rates, such as Siaya in western Kenya, reported a malaria prevalence of approximately 28.8% among children between 6 months and 14 years old^4,5^. Furthermore, the World Health Organization (WHO) reported that 546 out of every 1,000 people in Siaya county were infected with malaria in 2020, which required over KSh200 million (approx. 1.54 million USD) worth of medicine for treatment. Given the high burden of malaria in Kenya, particularly in high-risk regions such as Siaya, targeted intervention strategies have been implemented to mitigate transmission and improve public health^6^.

Standard malaria interventions include the widespread distribution of insecticide-treated nets (ITNs), indoor residual spraying (IRS), improved case management, the distribution of chemoprophylaxis and community-based surveillance programs^7^. Although these interventions have contributed to a decline in malaria transmission in susceptible human populations^8^, their long-term effectiveness is influenced by environmental, climatic, and socio-economic factors^9,10^. Among these malaria control measures, ITNs are the primary vector control method in many SSA countries because of their ease of large-scale distribution. A systematic literature review underlined that evidence is strong for the protective effect of ITN interventions in malaria prevention^11,12^, reflecting their proven effectiveness against night-biting mosquitoes. In 2021, a total of 17.9 million ITNs were distributed in Kenya by national malaria programmes (NMPCs), and 11.8 million were delivered by manufacturers^13^. Approximately 1 in 2 households (49%) in Kenya owned at least one ITN, and 29% of households have at least one ITN for every two people^14^. While NMCPs have made progress in increasing ITNs coverage, it seems that modelling approaches coupled with high-resolution climate, hydrological, and remote sensing information are proving to be powerful predictors^15^ and essential tools for guiding and optimizing malaria interventions.

Which modelling approaches can best capture key epidemiological challenges, such as identifying important disease drivers, disease hotspots, and seasonality of vector activity and disease transmission from climate variability/change to human interventions? Traditionally, the interaction between malaria interventions and climate variability has been examined primarily using statistical models. Statistical models utilise historical data to identify correlations between climatic factors (temperature, rainfall, and humidity) and socio-economic indicators (vulnerability, control effort estimates, including data on ITNs and IRS covariates, and malaria factors)^16,17^. They are essential for disease mapping, burden assessment, and resource distribution amid uncertainty. In this context, the Malaria Atlas Project uses Bayesian geostatistical models to estimate malaria burden across Africa. A notable example is the study by^10^, which employed statistical approaches to assess changes in malaria mortality in Siaya County from 2008 to 2019. The study found that environmental factors, particularly rainfall and vegetation cover, were significantly associated with malaria mortality, explaining up to 30% of the variation in mortality rates at the sub-county level. Interestingly, temperature was not identified as a significant predictor in their analysis. In addition, the study highlighted the role of non-climatic factors, showing that the scale-up of vector control interventions, such as ITN coverage, contributed to reductions in mortality of up to 20%. Yet, these effects varied geographically and were influenced by baseline transmission intensity and access to health services. Elsewhere, studies have independently assessed how variations in climate modulate the effectiveness of malaria interventions. In Zambia^18^, reported that a combination of changing rainfall patterns, potentially associated with El Niño events, and reduced vector control coverage explained much of the spatial and temporal variation in malaria prevalence from 2006 to 2012. Consequently, this highlights the inherent constraints of this approach to simulate the causal, nonlinear, and feedback-driven mechanisms that govern transmission dynamics, particularly in the context of changing climate and intervention scenarios.

Challenging the conventional approach, dynamical models provide a mechanistic understanding of how environmental factors, such as rainfall, temperature, soil moisture, land use and land cover changes, influence mosquito habitats, vector dynamics and ultimately malaria transmission. They are well-suited to conduct climate change risk assessments under different Shared Socioeconomic Pathways- Representative Concentration Pathways (SSP-RCPs) climate scenarios^19,20^. For example, the HYDREMATS model (Hydrology-Entomology and Malaria Transmission Simulator) has been used to simulate the formation and persistence of breeding sites in relation to hydrological conditions and landscape features^21^. Several studies have incorporated the effects of LLINs, IRS, treatment strategies, and behavioral adaptations into dynamic models of malaria transmission, often using compartmental or stochastic approaches^22,23^. These models have demonstrated how changes in intervention coverage, efficacy decay, and insecticide resistance influence transmission dynamics and intervention cost-effectiveness^24^.

Recent tools such as AnophelesModel have simplified the modelling of vector bionomics and interventions^25^. However, fewer studies have combined this intervention modelling with environmentally explicit hydrological or land surface models that mechanistically simulate the formation of breeding sites, the dynamics of surface water, or microclimate variability. The study by^26^ developed a mathematical model to evaluate the effectiveness of various malaria control strategies in endemic regions. The authors constructed a SEIR-type transmission model that incorporates the effect of interventions such as LLINs, IRS, and localized individual preventive measures. Through sensitivity analysis, they identified key parameters such as mosquito biting rate and the decay rate of intervention programs positively influence the number of new malaria cases, while treatment rate and intervention uptake negatively impact malaria prevalence. Following the recent tradition of linking pixels to people in human–environment systems, the VECTRI malaria model^27^ simulates spatially explicit malaria transmission dynamics (i.e. grid point-based) by using daily hydro-climate data (precipitation, 2 m air temperature and surface water) and demographic data to simulate both the vector and parasite life cycles, offering a powerful framework for dynamically assessing the role of environmental factors in malaria spread^15^.

While climate change is expected to alter malaria transmission, research has grown significantly in the field of climate-driven infectious disease modelling and intervention planning^28–32^. However, limited work has empirically explored how population dynamics, intervention strategies, and local hydro-climatic variability interact at high spatial and temporal resolutions to shape malaria transmission, forming a closed loop from atmospheric physics to human disease outcomes. This represents a critical research gap, especially in highly endemic and hydrologically dynamic regions such as Western Kenya Health and Demographic Surveillance System (HDSS). Our study addresses this gap by employing a hybrid modelling framework that combines physically based climate–hydrology simulations (WRF/WRF-Hydro) with a climate-sensitive disease model (VECTRI), and a parameter calibration method^33^ to simulate malaria transmission dynamics and optimize intervention (ITNs) effectiveness across varying environmental conditions. The key innovation of this work is not just predicting that a climate change will affect malaria, but explicitly modelling how it happens through the chain of physical hydrology and vector ecology.

In this paper, we move beyond traditional modelling approaches by simulating how convective rainfall events and localized warming captured by WRF generate transient surface water dynamics through WRF-Hydro, leading to the formation of ephemeral mosquito breeding sites. These environmental processes, together with temperature-driven parasite development, are coupled within an extended version of the VECTRI model that includes a novel intervention compartment to account for insecticide-treated bed net (ITN) coverage. This hybrid and spatially explicit framework enables the forecasting of hyper-local malaria outbreaks that are often overlooked by coarse-scale models, while also providing insights into the spatial variability in the effectiveness of vector control interventions. We adopt a two-step analytical approach. First, we use our coupled model system driven by climate and hydrological variables to simulate spatially and temporally resolved malaria transmission dynamics under ITN coverage. Vector-related parameters are then optimized using the best fit of simulated cases with reported data. Second, we validate the model outputs and quantify the effectiveness of ITNs across environmental gradients, thereby identifying thresholds for optimized intervention planning.

## Data and methods

### Study site description

**Fig. 1:**
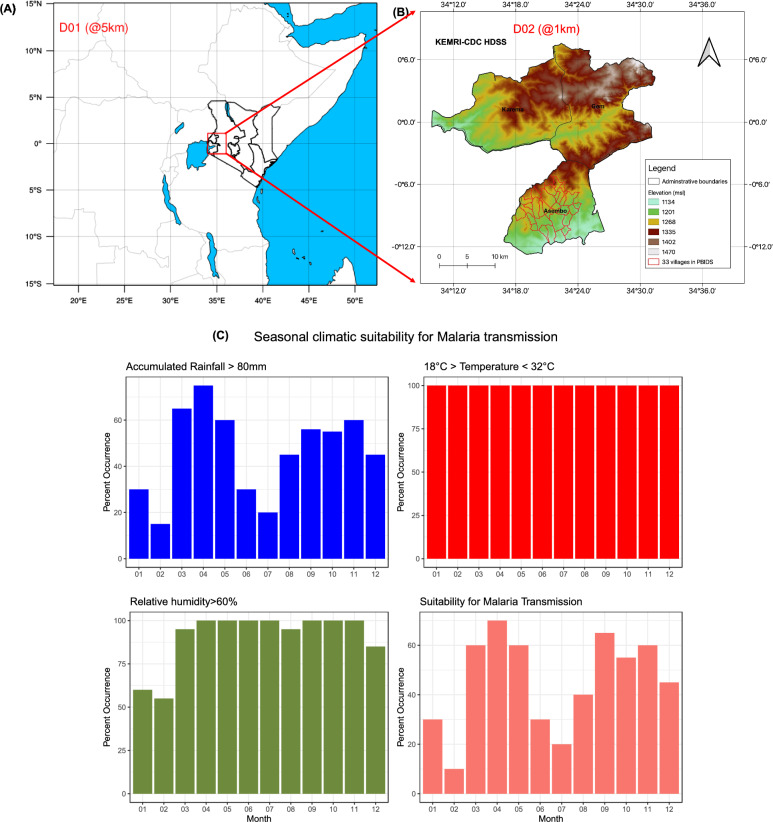
**A** The two WRF model simulation domains (d01, d02) nested within each other with horizontal resolution at 5 and 1 km, respectively. **B** The topography of the finest domain (d02) of the KEMRI-CDC health and demographic surveillance system (HDSS) area. **C** The observed percent occurrence of climate conditions (accumulated rainfall [> 80 mm], average air temperature [18$^\circ$C > T < 32$^\circ$C], relative humidity [>60%]) suitable for malaria transmission during 2007–2022.

The Siaya Health and Demographic Surveillance System (HDSS) is located in Siaya County. It spans Asembo, Gem and Karemo subcounties, respectively, in the western region of Kenya near the northeastern shores of Lake Victoria (Fig. 1A, B). Siaya lies between 0.26 $^\circ$S to 0.18$^\circ$S and 34.16$^\circ$E to 34.28$^\circ$E, covering an area of about 220 km2 characterized by a combination of rural and peri-urban settlements. Siaya is a malaria-endemic area, contributing to a significant malaria burden in Kenya^3,34^. The climate in Siaya is classified as tropical with two distinct rainy seasons. Long rains typically occur between March and May, while short rains fall between October and December. The annual average rainfall ranges from 1,000 to 1,800mm, with the highest rainfall occurring in areas closer to Lake Victoria^35^. Dry periods, between January and February, and June to September, often experience reduced agricultural productivity, influencing food security, household vulnerability and potential exposure to malaria^36^. The average annual temperature in the region ranges from 21$^\circ$C to 28$^\circ$C, with daytime temperatures occasionally rising above 30$^\circ$C during the drier months. The combination of warm temperatures and the presence of permanent water bodies provides optimal breeding conditions for Anopheles vectors (Fig. 1C), contributing to sustained malaria transmission throughout the year^37^. In recent years, Siaya has experienced the impacts of extreme weather events^38^. Episodes of intense rainfall have led to flooding, which increases mosquito breeding sites and exacerbates malaria transmission^39^. However, dry conditions often lead to an increased dependence of populations on stagnant water sources, which can still act as breeding sites for malaria vectors^40^.

### Satellite observations

The surface water maps were derived from Level-1 ground range detection Sentinel-1 data in VV polarization from the descending orbit with a 10-meter spatial resolution (later aggregated to model resolution at 50 m) from 2015 to 2020, providing a robust approach to high-precision hydrological and environmental monitoring. Sentinel-1 satellite data is often used in inundation mapping, due to its sensitivity to water. The data was preprocessed into calibrated, topographically normalized backscatter images. To classify individual Sentinel-1 scenes, a fully automated approach was then applied, using dynamic thresholds defined over permanent water bodies based on the Global Surface Water Explorer (GSWE^41^). The individually classified scenes are then combined into monthly surface water composites, in which false positives (mainly radar shadows) are removed by the use of the Multi-resolution Valley Bottom Flatness (MrVBF) index^42^ derived from the Copernicus Digital Elevation Model (DEM). This method is valuable for flood risk assessment, wetland monitoring, and water resource management, offering timely and detailed information on surface water dynamics. The soil moisture volumetric content daily dataset was derived from the Climate Change Initiative (CCI) of the European Space Agency (ESA^43^), at a spatial resolution of 0.25$^\circ$ (about 25 km × 25 km). This daily dataset integrates multiple active and passive microwave satellite observations, providing a consistent, long-term record of global soil moisture dynamics. This observed dataset is a valuable resource for hydrological modelling, climate studies, and the validation of land surface models.

### Malaria and bed net reported data

We validated the malaria modelling framework using a dataset previously published by the Kenya Medical Research Institute (KEMRI) in collaboration with the Centers for Disease Control and Prevention (CDC). The researchers implemented population-based infectious disease surveillance (PBIDS) across 33 HDSS villages in Asembo^10^. Data collection occurred monthly from 2007 to 2022, providing a continuous temporal record for analysis. This data set includes the number of reported malaria cases and the ITNs coverage. Analysis of ITN usage patterns reveals two distinct phases (see^10^) from 2008 to 2011, bed net use increased rapidly from approximately 75 to 93 (%/month), reflecting a major scale-up of ITN distribution likely associated with intensified national malaria campaigns. From 2012 onward, usage stabilised at high levels (93–98%), indicating sustained intervention coverage, with possible reinforcement through periodic mass distribution campaigns every three to four years. Despite minor fluctuations, the overall trend demonstrates consistently high coverage over the long term. Furthermore, HDSS population density data were used to address demographic variations and their possible influence on malaria transmission dynamics. Annual malaria cases were documented by the Global Burden of Diseases (GBD^44^), a collaborative network across 50 locations in Kenya from 2000 to 2022. The Kriging interpolation method^45^ was used to produce continuous spatial estimates of observational data at a resolution of 1 km for the Siaya HDSS region. This spatial interpolation method uses existing point data to estimate values at unsampled locations, resulting in a 1 km high-resolution dataset on an annual scale. This interpolated GBD dataset is used for model validation and parameter optimization.

### Driving climate and population data

We validated and evaluated our coupled simulations with various data sources from 2007 to 2022. Daily precipitation data (Pr) were derived from the Climate Hazards Group InfraRed Precipitation with Station data (CHIRPS^46^) at a resolution of 5 km, offering long-term rainfall estimates. Temperature data (Tas) was obtained from the Climate Hazards InfraRed Temperature with Stations (CHIRTS^47^) dataset, for the period 2007-2016 at the same 5 km spatial resolution. Furthermore, daily climate observations were improved by incorporating in situ weather station data (precipitation, air temperature, minimum and maximum temperature, and relative humidity) from the Kenya Meteorological Department to enhance the accuracy of atmospheric forcing inputs. The WRF output at 1 km resolution was initially resampled using the nearest neighbour method to match the CHIRPS 5 km resolution, thereby ensuring consistency in spatial comparison. We employed the t-test to statistically assess the significance of differences between simulated and observed climate drivers, including temperature and precipitation, across various periods. Significance was evaluated at the 95% confidence level (p < 0.05), ensuring that only substantial differences were included in the interpretation of model outcomes.

### Overall modelling chain description

Our approach is best described as a hybrid modeling framework combining the Weather Research and Forecasting (WRF) model version 4.0, widely used for weather and climate research at multiple spatial scales^48^, coupled with its hydrological component WRF-Hydro (version 5.2)^49^, and the VECtor-borne disease community model of ICTP, TRIeste (VECTRI^27^) model for simulating malaria transmission dynamics (see^15^), extended to include public health intervention dynamics. The WRF/WRF-Hydro modelling system simulates climate, surface, and subsurface hydrology variables at high spatial resolution. Model outputs include soil moisture and surface water accumulation, which are important variables in modelling mosquito breeding sites. The VECTRI component simulates disease transmission states within host and vector populations, along with the progression through all life stages of mosquitoes, from the eggs to larval and adult stages. The vector and parasite dynamics are driven by environmental inputs, such as rainfall, temperature, and the presence of water bodies, as provided by WRF/WRF-Hydro at resolutions of 1 km for the atmosphere and 50 m for the surface. The simulation core is coupled with a Genetic Algorithm (GA, see^33^), which employs evolutionary optimization to efficiently explore the high-dimensional vector-related parameter space and enhance model performance (see Table 1). Table 1 presents a comprehensive summary of the variables and parameters utilized in the model, highlighting their roles within the analysis. Each parameter is described alongside its definition, default value, best-fit estimate, and a key reference, enabling readers to understand the model’s structure and application while promoting transparency and reproducibility.

**Table 1:** List of parameters with their modified values in the VECTRI model set up for the Siaya region (“-” symbol indicates unitless).

Symbol	Parameters (definition)	Default	Best fit	Unit	Source
neggmn	Average number of laid eggs per batch that result in female vectors	80	119	eggs	^27,50,51^
rbiteratio	Biting rate	0.6	0.37	days	^27,50^
rhostclear	Clearance rate for non-immune adults	30	58	–	^27^
rpthost2vect_I	Probability of transmission from infected host to vector	0.2	0.84	–	^27,50^
rpthost2vect_R	Probability of transmission from *recovered* (R) vector to host	0.04	0.37	–	^27^
rptvect2host	Probability of transmission from vector to host	0.3	0.6	–	^27,50^
rrainfall_factor	Rainfall scaling factor	1.0	0.9	–	^27,51^
rbiocapacity	Maximum larval biomass per$m^2$of suitable water body	300	134	–	^27^
rlarvsurv	Base survival rate due to predation events	0.98	0.9	–	^27^
rimmune_gain_eira	Annual EIR required to gain full immunity	100	220	–	^27^
rimmune_loss_tau	e-folding timescale for immunity loss	365	309	–	^27^
rlarv_flushmin	Minimal daily larval survival (L1) rate after intense rainfall	0.4	0.9	–	^27,51,52^
rlarv_flushtau	e-folding factor for larval decay from flushing by rainfall	20	32	mm.day$^{-1}$	^27,51,53^
rbitehighrisk	Ratio of rate of bites for high risk to low risk	5	18	–	^27^
rlarv_tmax	Maximum temperature for larvae survival	37.0	36	$^\circ$C	^27,51,54^
rlarv_tmin	Minimum temperature for larvae survival	12	15	$^\circ$C	^27,51,54^
rlarv_eggtime	Time for egg hatching	1.0	0.8	days	^27,51,55^
rlarv_pupaetime	Time for pupal stages	1.0	1.4	days	^27,55^
rtgono	Threshold temperature for gonotrophic cycle (egg development in vector)	7	13	$^\circ$C	^27,51^
dgono	Degree-days to complete a full gonotrophic cycle	37.0	39	$^\circ$C$\cdot \ day^{-1}$	^27,51,55^
rtsporo	Minimum temperature threshold for sporogonic cycle (parasite development in vector)	18.0	15	days	^27,50,51^
dsporo	Degree-days to complete a full sporogonic cycle	111	87	$^\circ$C$\cdot \ day^{-1}$	^27,51,55^

### Model setup and calibration process

A two-nested domain was established at 5 km and 1 km, respectively, for the fully coupled and standalone WRF simulations spanning from 2000 to 2023. The configuration of the model included 40 vertical levels. The initial and boundary conditions were derived from the ERA5 reanalysis dataset, which operates at a 31 km resolution. The configuration of the WRF utilized in this study is thoroughly detailed in^15^. The innermost domain D02 was activated for WRF-Hydro, encompassing an area of 200 $\times$ 200 km² (Fig. 1A). The land surface model (LSM) was configured with a spatial resolution of 1 km, and the lateral runoff routing processes were addressed at a resolution of 50 m. The datasets used for WRF-Hydro were derived from the standard output of the WRF simulation for domain D02. The variables include precipitation, near-surface air temperature, humidity, wind, surface pressure, and both short- and longwave downward radiation. A coupling time-step of 1 hour was employed, facilitating the dynamic exchange of atmospheric and hydrological fluxes with high temporal resolution. The output frequency of the WRF/WRF-Hydro was set to a daily interval, enabling prompt and thorough evaluations of atmospheric conditions.

The VECTRI model includes parameters and thermal traits related to the *Anopheles gambiae s.s.* vector and the *Plasmodium falciparum* parasite. These parameters and traits are derived from field and laboratory studies; however, these parameters still have a certain level of uncertainty. Using the GA methodology from^33^, we calibrated VECTRI’s simulated malaria incidence against observed malaria incidence data. 22 parameters, including mosquito survival, bite rates, and larval development time, were perturbed to calibrate the malaria model outputs for the study site (see Table 1). The GA has been successfully employed to model malaria dynamics for a single location in Kenya^33^ and *Aedes albopictus* vector dynamics in multiple locations in Italy^56^. It distinguishes itself from a free parameter search by sampling and selecting parameter values within the bounds of their previously estimated uncertainty during the calibration process. GA is a powerful optimization method derived from the principles of natural selection. Potential solutions are developed through several iterations (“generations”) to identify the best set of parameters. This method is well-suited to address the intricate, non-linear relationships between environmental factors and malaria transmission. The GA is configured to iteratively perturb 22 parameters (Table 1) to minimize the root mean square error (RMSE) between simulated and observed malaria incidence for the whole district. The calibration process incorporates cross-validation to prevent overfitting. We utilized 80 ensemble members (n_ens) and 40 generations (n_gen), to explore the parameter space and drive model convergence towards an optimal configuration. In addition to the calibration step, we conducted a sensitivity analysis to evaluate the relative influence of each model parameter on the model’s outputs.

### Interventions compartment

ITNs reduce disease transmission through three main mechanisms: they act as a physical barrier to vectors, they increase mortality in adult mosquito populations, and they consequently reduce infection rates in humans. In the VECTRI model, malaria interventions are simulated using compartments for ITNs, IRS, and the sterile insect techniques (SIT). The ITN compartment is handled using either diagnostic (observational) or prognostic (dynamical) modes, depending on the model setting. In diagnostic mode (Case 1), the model directly assigns bednet coverage, $r_{\text {cover}}(x, y)$, (x denotes longitude and y latitude) based on the input field data, $r_{\text {input}}(x, y)$, and computes the proportion of treated nets, $r_{\text {treat}}(x, y)$, by applying a fixed treatment ratio, $r_{\text {treatratio}}$, to the coverage:

#### Diagnostic mode (input = total coverage rate)

1 $$\begin{aligned} r_{\text {cover}}(x, y)&= r_{\text {input}}(x, y) \ ,\end{aligned}$$

2 $$\begin{aligned} r_{\text {treat}}(x, y)&= r_{\text {input}}(x, y) \cdot r_{\text {treatratio}} \ . \end{aligned}$$

Conversely, in prognostic mode (Case 2), the model simulates the time evolution of net coverages and treatment levels based on a decay rate over time, $\tau _{\text {treat}}$, using the first-order explicit update

#### Prognostic mode (input = coverage rate per day per m^2^)

3 $$\begin{aligned} r_{\text {cover}}^{t+1}(x, y)&= r_{\text {cover}}^t(x, y) \cdot \left( 1 - \frac{\Delta t}{\tau } \right) + r_{\text {input}}(x, y,t) \ , \end{aligned}$$

4 $$\begin{aligned} r_{\text {treat}}^{t+1}(x, y)&= r_{\text {treat}}^t(x, y) \cdot \left( 1 - \frac{\Delta t}{\tau _{\text {treat}}} \right) + r_{\text {input}}(x, y,t) \ . \end{aligned}$$

Here, $\tau$ and $\tau _{\text {treat}}$ represent the characteristic decay times (lifespan) of bednet coverage and insecticide effectiveness, respectively, and $\Delta t$ is the model timestep. In the default case (absence of coverage information), both coverage and treated values are set to zero, disabling the effect of intervention in the model. As a compartmental susceptible-exposed-infected-recovered (SEIR) model, VECTRI incorporates ITN effects in both host-to-vector (Infected human to Susceptible mosquito) and vector-to-host (Infectious mosquito to Susceptible human) transmission pathways. For host-to-vector transmission, ITNs (rbednet_cover) primarily reduce the probability that a mosquito successfully bites an infectious human, thereby lowering the chances of the mosquito becoming infected. For vector-to-host transmission, ITNs reduce the effective biting rate, $z_{\text {bednet\_covfac}}$ and thus the infection risk (e.g., as estimated using the entomological inoculation rate, EIR). Both cases yield the following reduction factor:

5 $$\begin{aligned} z_{\text {bednet\_covfac}} = 1 - r_{\text {bite\_night}} \cdot r_{\text {bednet\_cover}} \ . \end{aligned}$$

## Results

### WRF-based results

**Fig. 2:**
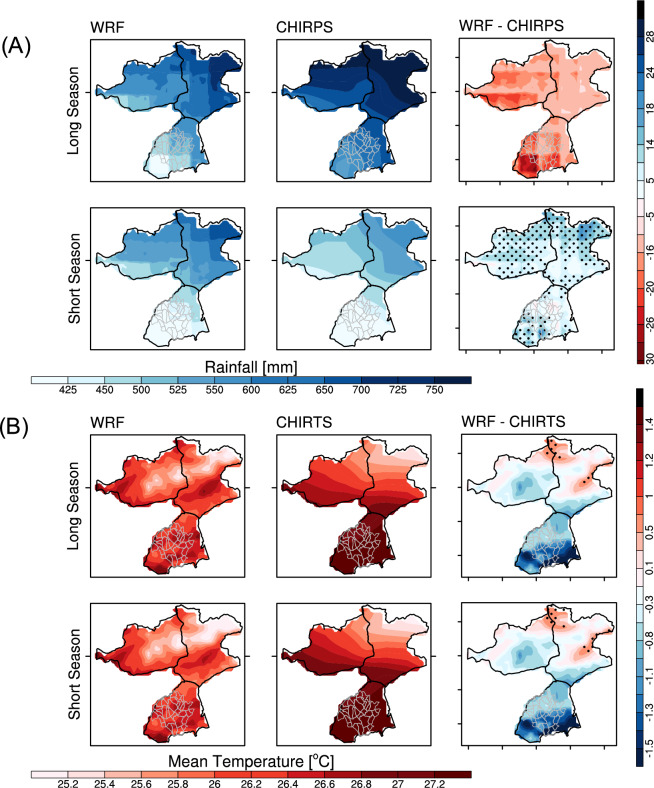
**A** Mean seasonal accumulated rainfall (mm) for the short (Oct–Nov–Dec) and long (Mar–Apr–May) rainy seasons based on WRF simulations (left), CHIRPS observations (middle) and mean rainfall bias (WRF minus CHIRPS, right). **B** Mean seasonal temperature ($^\circ$C) for the short and long rains for WRF simulations (left), CHIRTS observation (middle) and associated bias (right). Averages were calculated for the period 2007-2022, with dotted areas indicating significant differences at the 5% significance level < 0.05(p < 0.05).

The performance of the WRF model in simulating precipitation and temperature over the Siaya-HDSS is evaluated by comparing the spatial distribution of accumulated seasonal rainfall and averaged temperature with the CHIRPS (2007–2022) and CHIRTS datasets for the period 2007–2016 (Fig. 2). Consistent with previous studies assessing regional WRF performance in East Africa (e.g^46,57^.,), we observed a dry bias of up to 20 mm during the heavy rainfall period (March-May), and a wet bias of up to 15 mm is simulated during the short rainy season (October–December). Temperature biases demonstrate a consistent pattern across both rainy seasons, characterized by a cold bias of −1.5$^\circ$C in Asembo and a warm bias up to 1.2 $^\circ$C in the northern region, in agreement with prior regional evaluations^58^. The regional temperature biases underline the WRF model’s tendency to underestimate precipitation during peak rainfall periods slightly and to overestimate it during shorter seasons. The temperature bias also indicates that WRF generally simulates lower temperature values, especially in Asembo. A Mean Absolute Percentage Error (MAPE) of 15% for both rainfall and temperature over the whole region suggests a high level of prediction accuracy, consistent with commonly accepted thresholds in regional climate modelling (10–20%; e.g^59^.,).

### WRF-hydro-based results

**Fig. 3:**
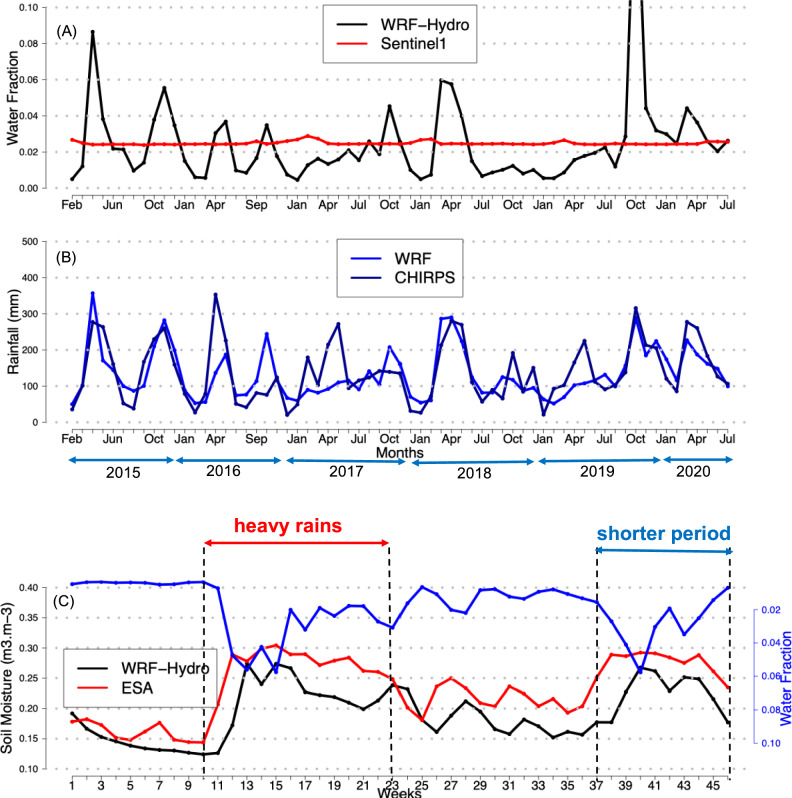
Monthly variations in **A** simulated WRF-Hydro and observed Sentinel-1 water fraction (%). **B** observed CHIRPS and simulated WRF monthly rainfall (mm). **C** Simulated (WRF-Hydro in black) and observed (ESA-CCI in red) mean weekly soil moisture (m3/m3) for the period 2015–2020. The blue solid line depicts simulated water fraction (%) by WRF-Hydro.

The water fraction (potential ponding water, in %) is analyzed using WRF-Hydro and Sentinel-1 data from February 2015 to July 2020. The WRF-Hydro simulations exhibit seasonal fluctuations, with well-captured wet and dry periods that follow the two main rainy seasons in the region (Fig. 3A). The variability of simulated water fraction closely follows the observed monthly rainfall distribution (Fig. 3B). This result indicates a strong coupling between precipitation and simulated surface water dynamics. Water fraction derived from the Sentinel-1 satellites rarely exceeds 2%, with the largest values observed in Asembo, in the southern part of the domain. Simulated water fraction values typically range between 1% and 3%, with larger values simulated in April 2015 (8%), November 2015 (5%), March–May 2018 (5.5%), and October–December 2019 (18%). We compare the mean weekly WRF-Hydro soil moisture outputs with observed ESA soil moisture data for the period 2015–2020 (Fig. 3C). This comparison seeks to provide deeper insights into the representation of hydrological conditions by the WRF-Hydro model, ensuring that the simulated infiltration, runoff, and subsurface water storage are consistent with satellite-based observations. The model reproduces seasonal soil moisture dynamics, with higher values observed during the long rainy season, consistent with precipitation-driven soil moisture variations. Previous studies have demonstrated model’s capability to accurately translate rainfall events into realistic soil moisture dynamics (Fig. 3) across diverse environments^15,60,61^, highlighting their relevance in the context of mosquito habitat suitability and malaria transmission dynamics.

Since the VECTRI model accounts for daily rainfall, the observed biases translating to minor daily differences (0.2 mm/day, Fig. 2) are unlikely to substantially affect the model’s representation of mosquito breeding site dynamics at the seasonal scale (Fig. 3). Moreover, malaria transmission exhibits a lag relative to rainfall, with peaks occurring approximately two months after rainfall events, suggesting that these small daily biases are unlikely to meaningfully alter the timing of transmission.

### VECTRI-based results

We first compare the spatial distribution of simulated annual malaria cases with the GHDx dataset over the Siaya HDSS for the period 2007-20. Then we focus on Asembo, where higher-quality observed malaria case data were available from 2007 to 2022.

#### Annual variations

**Fig. 4:**
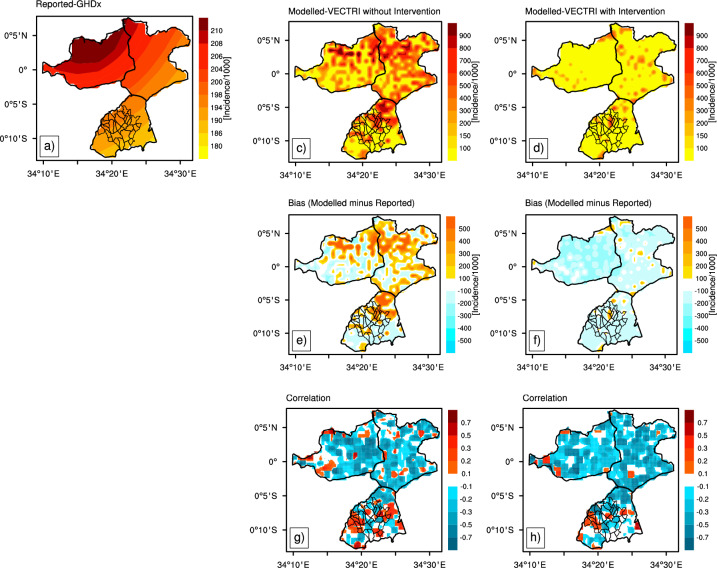
Observed (GHDx, (**a**)) and simulated (VECTRI) annual malaria incidence (per 1000) for the period 2007-20. VECTRI-simulated incidence is shown for simulations without (**c**) and with (**d**) ITN intervention. Corresponding annual malaria incidence biases (**e** & **f**) and Pearson correlation coefficients (**g** & **h**) are shown on the right-hand side panels.

Figure 4 compares observed (GHDx) and simulated (VECTRI, with and without the effect of intervention) mean annual malaria incidence for the period 2007–2020. In the upper row, the observed annual incidence shows a relatively homogeneous distribution, with slightly higher values in the western and central parts of the region, reaching a maximum of 210 cases per 1000 population. The spatial distribution of malaria cases from the GHDx dataset appears strongly correlated with rainfall patterns, reflecting the typical zonal gradient of precipitation in the region. Contrary to our expectations, simulated malaria incidence does not follow this zonal pattern and is more heterogeneous with hotspots over Gem and Karemo (see Fig. 1). This also indicate that rainfall has a positive effect on the incidence of malaria, but alone is an insufficient predictor and that local, mediating factors, particularly those governing the retention and pooling of water are the true determinants of transmission risk. It is this hydrological template that the VECTRI model uses to simulate the patchy distribution of vector breeding sites and subsequent malaria transmission hotspots. Such a heterogeneous pattern was observed in previous research conducted over Siaya^10^. VECTRI simulations, without the effect of ITNs, overestimate malaria incidence over most of the domain, particularly in the northern and southeastern regions, with positive bias values exceeding 400 cases per 1 (Fig. 4). In contrast, VECTRI simulations that include the effect of ITNs usage, show lower incidence values (Fig. 4, bottom row), closely aligning with observed estimates. Such improvement is reflected in the bias map, where the overestimation of malaria incidence by the model is significantly reduced (Fig 4). These results indicate that the inclusion of ITN intervention strategies modulates malaria transmission dynamics in the VECTRI model. Without the effect of ITNs, correlations are spatially heterogeneous, ranging from - 0.75 to 0.5, indicating limited model skill in reproducing the interannual variability in malaria incidence. When considering the effect of ITNs, correlation coefficients slightly increase, particularly over the southern part of the domain. The reduction in the mean bias and increase in correlation coefficient values when considering the effect of ITN intervention validate the new version of the VECTRI model and its potential applicability for forecasting and public health decision-making.

**Fig. 5:**
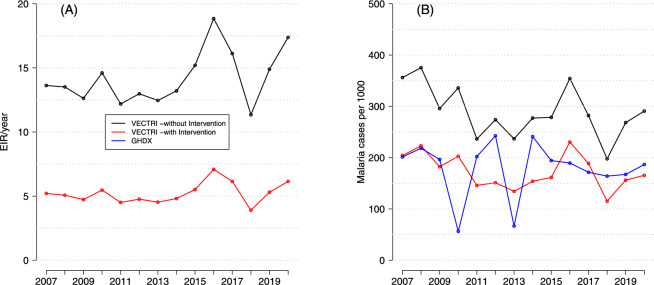
Annual variations in simulated **A** Entomological Inoculation Rate/EIR (infective bites per person per year) with (red line) and without (black line) the effect of ITN usage. **B** Comparison of simulated malaria incidence with GHDx observations (blue line) from 2007 to 2020.

Simulated annual Entomological Inoculation Rates (EIR) are presented in Fig. 5A. Figure 5B compares simulated malaria cases with observed data derived from GHDX for 2007-2020. Simulated EIR, without the effect of interventions (Fig.5A), range between 10 and 20 infectious bites per person per year. Simulated EIR values, with the effect of malaria control intervention, are lower, with an average of about 5 infectious bites per person per year over the study period. We also found a marked decline after 2017, further indicating the effectiveness of ITN-based vector control measures. Seasonal and inter-annual variations in simulated EIRs persist despite the incorporation of ITNs in the model. Simulated malaria incidence exhibits a similar pattern (Fig. 5B) in the Siaya HDSS. In the absence of interventions, simulated malaria incidence values are consistently large (between 200 and 400). When considering the effect of ITNs, malaria incidence values are lower and in better agreement with observed estimates from GHDx. A large interannual variability exists in reported GHDX malaria incidence values over the region. The better agreement between VECTRI simulations, which include the effect of ITN intervention, and GHDX data indicates that the malaria model effectively reflects the overall impact of interventions from 2010 onward. Discrepancies between simulated and observed malaria incidence at interannual scale remain. Such differences could be associated with other socio-economic factors impacting malaria burden^10^.

#### Monthly variations in malaria cases over Asembo-county

**Fig. 6:**
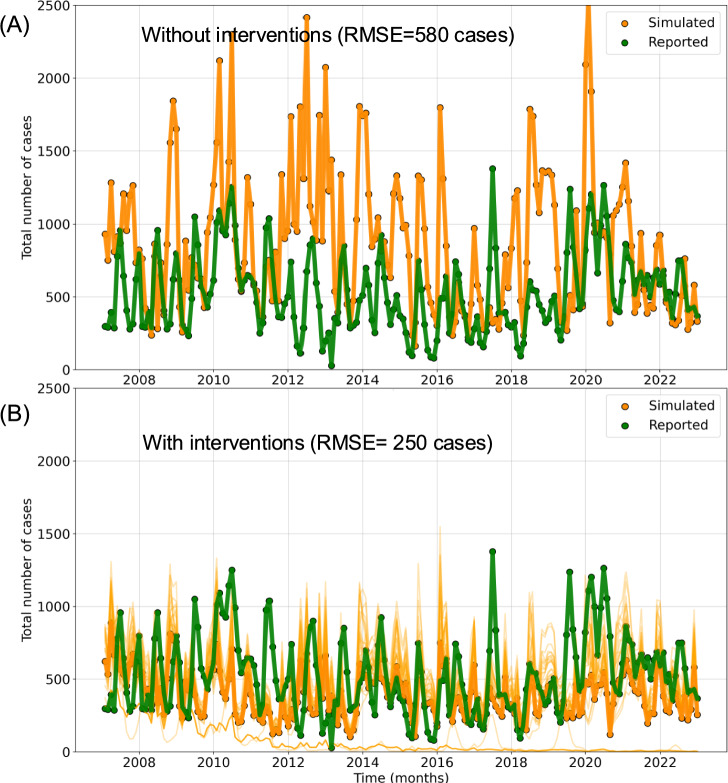
VECTRI-simulated model parameters vs. reported malaria incidence in Asembo: **A** without and **B** with the effect of ITNs for the period 2007–2022.

The simulated and reported number of malaria cases over Asembo are shown in Fig. 6. The number of reported malaria cases exhibits large interannual variability, with peaks and troughs reflecting seasonal malaria transmission dynamics. The^10^ forecasting model suggests that bed net use contributed to reductions with nuance in lags and interaction effects. The magnitude of the observed error (±100–150 cases/month) is likely within the inherent uncertainty of the malaria surveillance system itself^62^. Refining vector-related parameters (e.g., biting rates, ITN effectiveness, and environmental influences) during the calibration stage has increased the model’s ability to replicate observed malaria transmission dynamics. This improvement is crucial to improving the accuracy of the prediction, but some parameters may require further refinement.

#### Compound effect of hydro-climate factors on ITN effectiveness

**Fig. 7:**
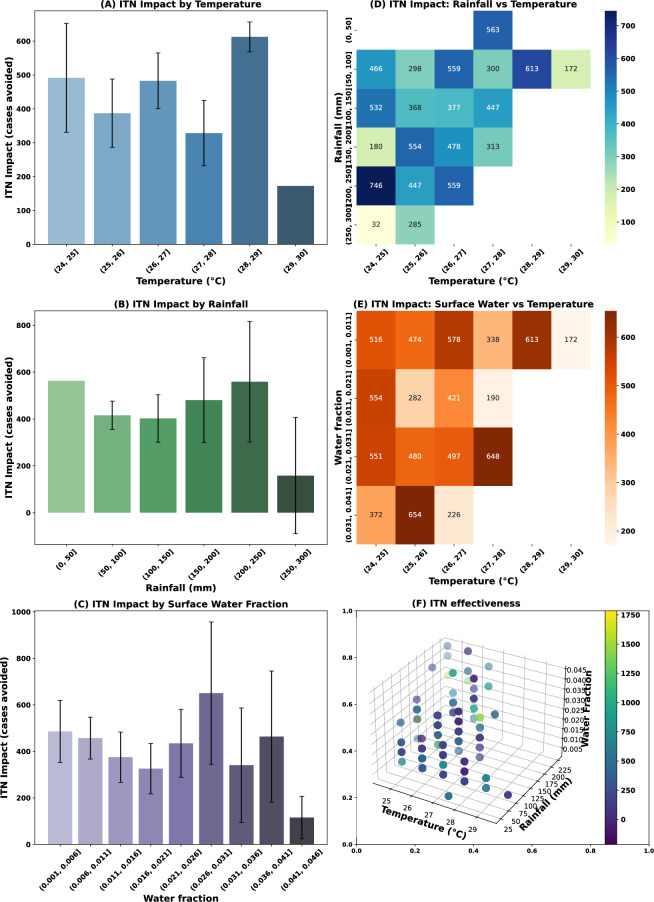
Environmental modulation of ITN impact across mean temperature ($^\circ$C), rainfall (mm), and surface water availability in Asembo for the period 2007–2022. Spatial averages were derived from gridded model outputs of malaria incidence, temperature, rainfall, and surface water fraction across the study domain. Environmental drivers were binned into discrete intervals: temperature (1$^\circ$C), rainfall (50 mm), and surface water fraction (0.01 unit). For each bin, we calculated the mean ITN-attributable case reduction and associated 95% confidence intervals. The bivariate heatmap was generated to assess the combined effects of temperature and rainfall/water fraction on ITN efficacy.

To quantify the impact of ITNs under varying environmental conditions, we computed the ITN coverage impact as the monthly difference in malaria cases between model simulations with and without ITN coverage (Fig. 7). Figure 7A shows that the effect of ITN generally increases with increasing temperature, peaking around 27–29$^\circ$C. This finding is consistent with the fact that both vector and parasite development rates accelerate within this thermal range, boosting transmission potential and thereby amplifying the effect of ITN use. Similarly, rainfall positively correlates with ITN impact (Fig. 7B), particularly within the 150–250 mm range, which corresponds to optimal breeding conditions for mosquitoes.

However, above 300 mm, this impact plateaus or declines slightly, possibly due to excessive rainfall flushing out breeding sites in the model. A nonlinear relationship between surface water fraction and ITN effectiveness is shown in Fig. 7C, with maximum values observed between 0.01 and 0.03. Moderate surface water presence supports stable mosquito populations that ITNs can effectively suppress. Conversely, very low or high water availability limits breeding, reducing both malaria transmission risk and potential gains related to the use of ITNs. The heatmaps in Fig. 7D and E show the combined effects of temperature, rainfall and surface water on simulated malaria cases. The largest ITN impacts (>600 cases avoided) occur where temperatures range between 26 and 29$^\circ$C, rainfall ranges between 150 and 250 mm (Fig. 7D), and the surface water fraction ranges between 0.01 and 0.03 (Fig. 7E). The optimal environmental envelope for ITN intervention lies in warm, moderately wet, and hydrologically active settings (Fig. 7F). These findings emphasize that the simulated effectiveness of ITNs is not uniform across space and time but is significantly modulated by climatic and hydrological variability.

## Discussion

While regional risk factors critically shape malaria transmission patterns, assessing their relative importance requires an integrated approach that combines spatial and temporal ground-based data with physically explicit modelling tools. Such an integrated approach is essential for understanding malaria dynamics, predicting trends, assessing the effectiveness of intervention methods, and guiding targeted control strategies and elimination efforts. To this end, we developed a high-resolution dynamical modelling framework that integrates WRF, WRF-Hydro, and VECTRI at 1 km spatial resolution in a malaria-endemic region of western Kenya.

Our results show that WRF (Fig. 2) slightly over- or underestimates precipitation during the long rainy season (roughly +25 mm in March–May) and the short rainy season (about −15 mm in October-December), which is consistent with previous regional climate modelling studies^58,63^. Simulated surface air temperature shows a better agreement with CHIRTS data, with a small negative bias. The findings align with known challenges in regional climate modelling over East Africa. ^57^ attributed similar WRF model biases to difficulties in simulating the precise dynamics of mesoscale convective systems during heavy rainfall events and the model’s sensitivity to convective parameterization schemes. Our study, which uses a different model configuration and focuses specifically on Siaya, corroborates this broader pattern. This consistency suggests that the bias is not merely a product of our specific setup but a more general characteristic of the model in this region’s complex topographic structure^58,64^. It should be noted that CHIRPS and CHIRTS prove especially valuable in regions with sparse or inconsistent weather station networks, though some bias may persist in high-elevation areas, which should be considered when interpreting model performance. In general, WRF-Hydro can account for observational data, such as rainfall datasets to reduce bias and spatial displacement, but the data at 1 km is not available.

Surface hydrology also plays a critical role in providing mosquitoes’ breeding sites, particularly in areas with temporary water bodies and slow-draining wetlands. Discrepancies between surface water maps generated by the WRF-Hydro model and those derived from Sentinel-1 observations (Fig. 3A) might be attributed to the fundamental representational differences inherent to each dataset. First, WRF-Hydro’s grid-cell averaged representation of soil saturation and water depth results in a spatially smoother and more diffuse wetness signal compared to the observations. Second, Sentinel-1 data is valuable, but could have limitations in different eco-regions due to caveats associated with temporal sampling^41^, vegetation interference^65^, hydrological variability^66^, and soil and land surface properties^41^. These inconsistencies are particularly large in Siaya county, a region that experiences two rainy seasons and dense vegetation cover. These factors likely contributed to Sentinel-1’s limitations in accurately detecting surface water dynamics. Sentinel-1 is a radar-based synthetic aperture radar (SAR) system that takes measurements every five days, but sensor limitations and environmental factors restrict its ability to capture transient and small-scale water bodies^65^.

In contrast to our previous work in Nouna^15^, a semi-arid region of Burkina Faso characterized by a single rainy season and sparse vegetation, Sentinel-1 well captured surface water extent, likely due to fewer obstructions from dense vegetation and a reduced presence of transient water bodies. Indeed, the interplay of factors influencing vector transmission differs significantly across regions, rendering generalizations potentially misleading. The WRF-Hydro model accurately captures the seasonal cycle of ponding and reproduces major hydrological extremes (2015, 2018, and 2019, Fig. 3B) in consensus with the findings of existing literature^67–69^, demonstrating strong agreement with observed rainfall, soil moisture patterns. This improved representation of hydrological features is essential to identify high-risk zones for malaria transmission, particularly in low-lying areas where water stagnation can last for 7 to 21 days^70^. Our model captures the synoptic-scale hydrologic conditions that must be met for widespread mosquito breeding to occur. When our model indicates sustained surface saturation in a region, it reflects a landscape where the topographic, soil, and meteorological conditions are conducive to creating the numerous small-scale habitats that^71^ so effectively model. However, unlike Asare’s work, which focuses primarily on static or empirically derived habitat distributions, our coupled WRF/WRF-Hydro–VECTRI framework dynamically simulates the physical processes governing water accumulation and drainage over complex terrain. This allows us to resolve the transient and spatially heterogeneous nature of breeding site formation in response to short-term convective rainfall and land surface feedbacks factors that are often oversimplified or omitted in purely statistical or spatial correlation models.

While environmental factors create suitable conditions for malaria transmission, human interventions can substantially modify transmission dynamics. This study incorporates the effect of ITNs into the VECTRI malaria model to assess the impact of control measures on transmission risk. The effect of ITN intervention in simulations significantly reduces annual entomological inoculation rates (EIR) up to 57%, leading to more realistic values with respect to observed reported case estimates in Siaya. In^31^ model simulations, the high ITN usage was necessary to achieve a substantial decrease in clinical incidence in high-EIR settings, up to levels approaching 80–90%. While the Kenya Malaria Strategy^72^ reports county-level incidence rates ranging from 200 to 450 cases per 1,000 population in high-transmission areas, the GHDx dataset for the Siaya HDSS reports a lower maximum of only 210 cases per 1000 (Fig. 4). This fundamental discrepancy suggests that uncertainty (under-reporting biases) on reported malaria cases, spatial interpolation of local hotspots or aggregation methods of health facility-based reporting used in the GHDx dataset may under-represent malaria burden in low-level high-risk regions. This creates a validation gap where models are forced to be evaluated against data that is fundamentally incompatible with their inherent resolution, masking potential inaccuracies in the simulated processes. VECTRI simulations that include the effect of interventions yield more consistent estimates with respect to values reported in the national policy report^72^. This divergence highlights the need to critically evaluate global burden datasets when applied at subnational scales, especially where localized surveillance data suggest significantly higher transmission intensity.

In Asembo, malaria incidence simulated by VECTRI without accounting for ITN coverage significantly overestimated observed cases, while including intervention data reduced the model error by nearly 41% (Fig. 6). Comparative modelling studies in coastal regions of Kenya (50%) have reported lower reductions in incidence, around 32%, likely due to regional differences in ITN ownership and usage rates. Given that ITN coverage and utilization tend to be higher (63%) in the Lake Victoria basin (where Asembo is located) our findings align with the hypothesis that the effectiveness of ITNs is not only intervention-dependent but also shaped by local environmental and behavioral contexts.

It is well documented that health-facility–based malaria surveillance systems suffer from underreporting, delayed diagnosis, and reporting biases^73,74^. Moreover, modeling studies (e.g^33^.,) have shown that input and parameter uncertainties can introduce substantial variability into simulated incidence. Therefore, an error of ±100–150 cases/month between our simulations and reported data likely lies within the combined uncertainty envelope of both the model and the surveillance system itself. This result supports earlier findings that vector control strategies, particularly ITN deployment, are essential for realistically capturing malaria dynamics in high-transmission settings^10^. Field studies focusing on Anopheles gambiae & Anopheles funestus in Africa show that ITNs reduce exposure to nighttime bites by 50–80%^75,76^. Despite the large reported bednet coverage in Siaya (up to 98%) the region remains endemic, raising questions about the consistency and effectiveness of actual ITNs usage across the population. High ownership does not necessarily equate to high utilization, and behavioral, socio-economic, and logistical factors may limit the protective impact of ITNs. Mathematical modelling by^77^ suggested that to achieve the WHO target of a 90% reduction in malaria burden and progress toward malaria-free by 2030^78,79^, recommended interventions must reach and maintain at least 90% effective coverage. This underscores the importance of not only distributing ITNs, but also ensuring their proper and sustained use across all demographic groups.

To assess the robustness of our simulations, we compared our modelled ITN-attributable reductions (Fig. 7) with recent empirical and modelling studies across sub-Saharan Africa. A quantitative comparison with existing literature reveals both consistencies and divergences. For instance^80^, report that the entomological efficacy of ITNs tends to decline as insecticide resistance increases, underscoring that the effective reduction in vector abundance may be lower than theoretical maxima. Similarly^81^, demonstrate that under higher ambient temperatures and net decay, ITN effectiveness can fall considerably over time, aligning with the lower case reductions we observe in bins with high temperature and low rainfall or water fraction. In Kenya^10^, quantified spatial heterogeneity in the effect of ITN usage by incorporating climate variables and found that parasite prevalence reductions were not uniform across counties, suggesting that environmental context modulates ITN impact^82^. found an empirical protective effect of 32% in coastal malaria zone of Kenya, which is classified as a malaria-endemic area. ITN users, which is a useful benchmark for interpreting our modelled case reductions. Our modelled reductions fall within plausible bounds when considering these field and modelling studies, especially in moderate climate bins; nevertheless, discrepancies in extreme climate bins may reflect the intensifying role of resistance, net decay, or unmodeled behavioral and ecological factors.

The East African Community, including countries such as Kenya, aims to achieve malaria-free status by 2050^72^. Persistent challenges such as the high costs of interventions, gaps in surveillance, limitations in vector control, and restricted access to diagnosis and treatment in health facilities still remain^78,83^.

The coupling of the WRF, WRF-Hydro, and VECTRI models at high spatial resolution represents a novel hybrid approach to bridging the gap between atmospheric, terrestrial, and epidemiological modelling, offering valuable applications for developing malaria early warning systems and public health decision-making. Our hybrid framework is better at simulating the “why” and “when” of malaria outbreaks based on first principles of biology and climate, rather than just the “where” based on past correlations. However, a systematic deviation between simulated and reported cases still remains, particularly during transmission peak periods, indicating that further refinements in climate input data, intervention modelling, or human behaviour factors could further improve the accuracy of the model. VECTRI model performance is largely affected by accurately parameterizing thermal processes within the model. Our sensitivity analysis revealed that parameters related to temperature exert the greatest influence on the model outputs. Specifically, variations in parameters governing mosquito development rates, parasite incubation periods (degree-days for parasite development, threshold temperature for parasite development), and mosquito survival (threshold temperature for egg development in the vector, and maximum and minimum temperature for larvae survival), all of which are temperature-dependent produced the most substantial changes in VECTRI-simulated malaria incidence (see Table 1). Our results require further confirmation, initially after calibrating with the ITNs to gauge the effectiveness as a function of the base temperature by going from the cold borderline to the peak of the borderline and then addressing the vector Aedes albopictus responsible for dengue and chikungunya viruses. Future work could expand the model to include human mobility patterns and socio-economic determinants, which are known to influence transmission heterogeneity but are not yet captured in the current framework.

## Conclusion

This study presents a high-resolution hybrid climate–hydrology–malaria modeling framework that integrates environmental processes, mosquito ecology, parasite biology, and intervention dynamics to simulate malaria transmission in western Kenya Health and Demographic Surveillance System (HDSS) villages (2007–2022). The model reproduces observed epidemiological patterns with high fidelity and confirms the essential role of bed-net use in shaping transmission intensity. Agreement with reported clinical cases (mean error ±100–150 cases per month) falls within the range of surveillance uncertainty across Africa, and inclusion of ITN coverage reduced simulated incidence by 41%. These results demonstrate that explicit representation of intervention dynamics is essential for converting theoretical risk into actionable disease forecasts. Despite this strong validation, challenges persist in obtaining bias-free environmental inputs and in parameterizing fine-scale hydrological and biological processes under limited entomological and epidemiological data. Addressing these constraints through multi-site analyses (using data from other HDSS locations or comparable platforms with varying endemicity and socio-economic contexts) and the intervention module to simulate additional strategies such as indoor residual spraying (IRS) could strengthen the model’s generalizability and reliability. The findings of this work point to several critical directions for future research. In particular, the greatest potential of this physically grounded framework lies in its ability to simulate scenarios of environmental change and assess their implications for malaria transmission. Beyond methodological advances, the framework also provides a valuable tool for exploring the co-benefits of climate-adaptive and nature-based solutions (NbS) for malaria control, such as landscape restoration initiatives that mitigate vector breeding through hydrological modification.

## Data Availability

Climate Hazards Group InfraRed Precipitation with Station data (CHIRPS, Funk et al. (2015)) information and data access can be obtained at https://www.chc.ucsb.edu/data/chirps. The minimum and maximum CHIRTS-daily temperatures can be downloaded from https://data.chc.ucsb.edu/products/CHIRTSdaily/v1.0/(Funk et al., 2019). The gridded population density data sets can be accessed from https://sedac.ciesin.columbia.edu/data/set/gpw-v4-population-density-rev11 (Doxsey-Whitfield et al., 2015). The Malaria and bed net data used in this study are available from the Kenya Medical Research Institute (KEMRI) CDC Data Access/Ethics Committee for researchers who meet the criteria for access to confidential data. Any data requests may be sent to the respective steering committee through Dr. Stephen Munga (Smunga@kemri.org). Satellite-based Sentinel-1 data information and access can be found at https://sentinel.esa.int/web/sentinel/sentinel-data-access. All analyses and figures were drawn in the R Foundation for Statistical Computing version 4.2.2 Platform (R Core Team, 2022), Python version 3.9 (Python Software Foundation) and NCL version 6.6.2 (NCAR Command Language).
